# Our Immunity from Plague

**Published:** 1897-03

**Authors:** 


### [redacted] OUR IMMUNITY FROM PLAGUE.

While all Europe is watching with unfeigned dread the progress of the Bombay epidemic of malignant polyadenitis,[1] we in America have only that vague dread that comes from the uncertain but improbable. Since the discovery of America there have been many epidemics of this terrible scourge in different parts of Europe, but at no time has it succeeded in crossing the Atlantic to our eastern shores, or the Pacific to our western. In 1852 it succeeded in reaching as far across the Atlantic as the Canary Islands, and lately it crossed the Chinese Sea to Formosa. Encouraging as this seems, much of its satisfaction is stolen from it by the fact that modern steamships and modern commerce have annihilated distance, making Europe and even India our next-door neighbors as compared with what they were less than half a century ago. While wide oceans once were an effectual barrier to its progress, we are not so sure that they still continue to be such. That they aid in our protection can not be doubted, but to what extent or how they do so is uncertain. The antiseptic action of long exposure to sea air and sea conditions may degrade if it can not destroy the virulence of the microbes.

1 This exceedingly appropriate name has been given to the bubonic plague by James Cantlie, M. B., F. R. C. S., of Charing Cross Hospital Medical School, London.

That even short distances by sea possess a marked staying power to the plague, is seen in the fact lately pointed out by James Cantlie, M. B., in his lecture, in London, on "The Spread of Plague." The distance from Hong-Kong to Macao is thirty miles. It took ten months to carry the disease from the one port to the other, although steamers, Chinese junks, and fishing boats traveled daily across and carried thousands of fleeing citizens who were seeking to escape its ravages. Other cases of the same kind have been observed in the past. In the Macao case, it is not even sure that it went by sea at all, some persons believing that the virus was carried overland from Canton, or that it spread like a ringworm through the soil from this place. In the face of such facts as these, there need be no great fear on our part of its troubling us. It took so many months to go so short a distance, when no artificial obstacles of any kind were placed in its way and when conditions were such as to favor its transmission to a remarkable degree, it is not all likely that it will find its way across the Atlantic in other than isolated and non-malignant cases. In London, two bad cases have reached the Seaman's Hospital. In Marseilles, several doubtful cases have occurred among sailors direct from India. No doubt, Macao had such cases long before there was any sign of its becoming epidemic.

A study of the means by which it is transmitted from person to person, will shed some light on the way it travels from place to place. In the very height of its ravages, doctors come and go without being attacked. Nurses who refrain from close personal contact with patients, do not suffer. The handling and fondling of sick babies or children gives it. As in tuberculosis, the first and in some cases the only immediate victims are parents, children, husbands, wives, uncles, cousins, aunts, grandparents or lovers—all those who are liable to come in close, continuous personal contact. Many cases, of course, occur where it is difficult or impossible to trace their source. These are pretty likely to have been produced by some infected article of food. In rat-eating China or famine-stricken India, where the very hungry might be expected to eat rats, it would, of course, be a simple matter to trace the origin of the disease, knowing as we do that these rodents are among the first victims. Pigs that have had their freedom, and in their hunger devoured rats, mice, or other infected animals that have died of plague, would become disseminators of the disease among all except those whose religion forbade their eating pork. Pigeons that when hungry have developed carnivorous traits, or that have sought food that has become mixed with the feces of victims, will, when eaten, give the disease. Flies that have swarmed among filthy discharges and then walked over the food people have been eating, would likewise give it

It is quite likely that barnyard fowls may take the disease and transmit it, but there is no evidence of such cases. The diplobacterium has been found in the feces, in the blood, and in the mouths of victims. It develops on contact with moist mucous membranes, by contact with abraded surfaces, or by being swallowed in food. Some soldiers that were set to work cleaning out an infected and filthy abode, after the death of its inhabitants, were attacked. The dried feces in the dust-laden air gained access in sufficient amount to give them the disease. Here we see how the immunity of the individual may play an important part. A definite amount of the virus is needed to overcome the power of resistance each person possesses. The long contact necessary in most instances is probably due to this. It takes such time for them, under such conditions, to take in enough germs to give them the disease in its virulent form. The multiplication of cases in narrow, close quarters increases the amount that is likely to be absorbed in a given time, and likewise increases the virulence of the germs. When no pains are taken by them to remove from their rooms excreta or filth of any kind, this, too, multiplies the chances of producing violent instead of ambulant cases. To force cleanliness, to scatter the victims into well-aired rooms away from the healthy, and to avoid close personal contact, seem to be nearly all that is necessary to keep this disease from spreading in its malignant form. Those who fly from stricken cities and scatter themselves far and wide, evidently weaken the contagion by the act of diffusion alone. Pestis minor occurs in their wake, but not for long periods of time do they, by concentrating themselves amid filth again, seem to be attacked by the malignant form. In all the European epidemics of the past, filth and crowding seemed to be at the bottom of the trouble. The chronicles of those times show that England was in a horrid state of filth. The poor were huddled together in houses with single rooms. The bare earth constituted their floors. They had no closets and attended to the wants of nature in their living apartments, merely covering up the filth with straw. They stood, sat, and slept on this straw. Soldiers sometimes were sent to clean their floors for the sake of the nitrogenized filth, from which they extracted nitrates to be used in the manufacture of gunpowder. Such were the horrid conditions that gave rise to the fierce attacks of black plague. We have nothing like it in America. The worst quarters of New York or San Francisco are far above such conditions.—*American Medico-Surgical Bulletin.*

